# Host Immune Response in Chronic Hepatitis Delta: Implications for Pathogenesis and Therapy

**DOI:** 10.3390/pathogens14080828

**Published:** 2025-08-21

**Authors:** Arshi Khanam, Abutaleb Ameer, Poonam Mathur, Cihan Yurdaydin, Shyam Kottilil

**Affiliations:** 1 Division of Clinical Care and Research, Institute of Human Virology, University of Maryland School of Medicine, Baltimore, MD 21201, USA; pmathur@ihv.umaryland.edu (P.M.); skottilil@ihv.umaryland.edu (S.K.); 2 Department of Surgery, George Washington University School of Medicine and Health Sciences, Washington, DC 20037, USA; aabutaleb@mfa.gwu.edu; 3 Department of Gastroenterology & Hepatology, Koç University School of Medicine, Istanbul 34450, Turkey; cyurdaydin@ku.edu.tr

**Keywords:** chronic hepatitis delta, host immune response, cirrhosis, hepatocellular carcinoma

## Abstract

Chronic hepatitis delta (CHD) represents the most severe form of viral hepatitis due to rapid disease progression towards liver cancer, leading to high morbidity and mortality. Hepatitis delta virus (HDV) can only infect individuals who are infected with hepatitis B. So far, there is no cure or vaccine for HDV. Existing treatment options, including pegylated interferon-α and hepatocyte entry inhibitors, offer limited efficacy. Emerging therapeutic strategies are focused on targeting various steps of the HDV life cycle or enhancing the host immune response to promote viral elimination. A defective antiviral immune response is increasingly recognized as a culprit for HDV persistence; however, the precise immunological mechanism associated with disease progression and pathogenesis has not been well defined. This review provides an update on the current understanding of host immune response in CHD, highlighting its role in both disease pathogenesis and viral clearance. A deeper understanding of these immune correlates may lead the way to novel treatment strategies, including immunotherapies targeting host immune response that can be used in combination with other antiviral therapies to achieve more effective and durable treatment outcomes.

## 1. Introduction

Hepatitis delta virus (HDV) is the smallest known hepatotropic human RNA virus (35–36 nm), belonging to its own separate genus “Deltavirus”. HDV causes the most severe form of chronic viral hepatitis due to rapid progression towards end-stage liver disease, such as cirrhosis, hepatocellular carcinoma (HCC) and liver failure, leading to high mortality [1,2]. HDV can infect only those individuals infected with the hepatitis B virus (HBV), as its genome is too small to encode the replicative enzymes and envelope proteins required for its own replication and assembly. Therefore, it depends on HBV to provide the envelope proteins for cell entry and to support its propagation in hepatocytes; hence, it is recognized as a “satellite virus” [3,4]. HDV genome contains a single open reading frame that produces two isoforms of a delta antigen, including a small delta antigen (SDAg) and a large delta antigen (LDAg). The SDAg is essential for HDV replication, while the LDAg interacts with hepatitis B surface antigen (HBsAg) for packaging of HDV RNA and shares the same hepatocyte receptor as HBV for viral entry [5]. HDV affects approximately 5% of individuals infected with chronic hepatitis B (CHB) and occurs either as an acute coinfection by HBV and HDV or as a superinfection in individuals infected with CHB [6]. Remarkably, while concurrent infection with HBV and HDV results in the clearance of both viruses in the majority of the patients, HDV superinfection leads to persistent coinfection in approximately 90% of cases [7]. Injectable drug users are at a great risk of getting HDV superinfection [8]. However, in industrialized Western countries such as Western Europe, Australia, the USA and Canada, immigrants from HDV endemic regions now represent the primary at-risk group.

Effective antiviral treatments are available for other viral hepatitis, such as hepatitis B and C; however, such therapies are lacking for HDV infection, imposing substantial risk for severe outcomes. The reason why conventional antiviral therapies are not effective for HDV infection is that HBV and HDV replicate through different mechanisms [9]. Though HDV depends on HBsAg for the formation of viral particles, it replicates independently using the host’s ribonucleic acid (RNA) polymerase, not HBV’s replication machinery. HBV antivirals mostly target HBV deoxyribonucleic acid (DNA) synthesis and reverse transcription, which do not affect HDV’s RNA genome replication. In addition, even when HBV DNA is suppressed, persistent production of HBsAg enables HDV to continue assembling infectious particles. Consequently, it imposes a minimal impact on HDV replication and disease progression, necessitating distinct treatment approaches targeting HDV-specific pathways. While patients with HDV infection are often treated with pegylated interferon alfa (IFN-α), the response to treatment is reasonably poor and HDV clearance is rarely achieved [10]. Also, the lack of suitable experimental systems to study the HDV life cycle confines the development of HDV-specific drugs [11]. Therefore, a detailed understanding of both HDV virology as well as HDV-specific host immune response is critically required for the development of new therapeutic interventions.

In this review, we have focused on the various immunological mechanisms that are associated with either viral clearance or are involved in viral persistence, disease progression and severe outcomes in these patients, with the aim of enhancing our understanding of chronic hepatitis delta (CHD) infection. This knowledge might help in the development of new global strategies for therapeutic interventions.

## 2. Role of Innate and Adaptive Immune Response Against HDV Infection

Effective control of viral infection requires coordination between the innate and adaptive immune response. Innate immunity rapidly recognizes the viral particles, including nucleic acid and proteins, by inducing an antiviral state through the production of type I interferons. This not only promotes the killing of the virus-infected cells but also supports the development of adaptive immunity via the production of cytokines and chemokines [12,13]. Subsequently, the adaptive immunity functions through the maturation and expansion of T and B cells that specifically recognize the infectious agents and eliminate them by antiviral mechanisms. This further generates an immunological memory response that protects against subsequent infection with similar pathogens [14,15].

Generally, the host’s innate and adaptive immune systems play a crucial role in eliminating HBV infection; however, HBV has evolved and established strategies to escape the host immune system, resulting in persistent infection [16]. Infection of CHB patients with CHD may further compromise immunological response and impair intrahepatic immunity by driving rapid viral replication, thereby promoting viral persistence and rapid disease progression towards end-stage liver disease, resulting in severe clinical outcomes.

### 2.1. Pattern Recognition Receptors

One of the earliest events in HDV infection is the activation of pattern recognition receptors (PRRs) on liver cells. The detection of the pathogen-associated molecular patterns (PAMPs) by PRRs such as endosomal toll-like receptors or cytosolic RIG-I-like receptors (RLRs) triggers innate immune responses in the infected individuals [17,18]. The RLRs constitute three PRRs, including retinoic acid inducible gene I (RIG-I), melanoma differentiation associated gene 5 (MDA5) and laboratory of genetics and physiology protein 2 (LPG2), where RIG-I and MDA5 are typical PRRs, while LPG2 is involved in the regulation of RIG-I and MDA5-mediated signal transduction [19,20]. Upon pattern recognition, RIG-I and MDA5 interact with the mitochondrial antiviral signaling protein (MAVS) that subsequently triggers the activation of the transcription factors, including interferon regulatory factors 3 and 7 (IRF3/7) and nuclear transcription factor-κB (NF-κB). This cascade drives the upregulation of proinflammatory and antiviral genes, thereby inhibiting viral replication and triggering a robust immune response that facilitates virus control/elimination [21,22].

Being an RNA virus, HDV is identified by several PRRs, particularly RNA sensors like RLRs [23]. While the exact nature of HDV-specific molecular patterns activating PRRs has not been well defined, partial dependence on RLR signaling has been reported [24,25]. Primary human hepatocytes and non-transformed differentiated HepaRG cells (dHepaRG) express several innate immune receptors comprising TLRs and lymphotoxin β receptor (LTβR), which upon activation can induce various inflammatory and anti-inflammatory responses [26,27]. Stimulation of hepatocytes by TLR1/2 ligands (Pam3CSK4) and LTβR (BS1) inhibits HDV replication by limiting the amplification of intracellular HDV RNA through the induction of the NF-κB pathway [28]. Additionally, these ligands negatively affect HDV progeny release and competently decline their specific infectivity, restricting viral spread in vivo settings, a finding that signifies the role of PRRs during HDV infection. In addition, the dysregulation of metabolic pathways that are critical for HDV propagation may further decline viral replication. These findings might have potential significance as TLR agonists such as GS-9688 (TLR8 agonist) are currently under clinical evaluation for HBV treatment, and preliminary findings are quite promising [29,30]. Similarly, the TLR1/2 agonist Pam3CSK4 can be considered for therapeutic implications [28] and, in fact, may offer broader efficacy than TLR8 agonists, as TLR1/2 receptors are present on the hepatocytes, allowing the direct action of the ligand on these cells. In contrast, TLR8 receptors are present only on immune cells; so, their agonists act exclusively on immune cells rather than hepatocytes. There are certain limitations with Pam3CSK4, as it cannot be given orally like GS-9688 and would require targeted delivery to the liver to prevent ligand degradation and limit potential adverse effects from systemic exposure. Nonetheless, this can be circumvented by using nanoparticles (NPs) to target liver-specific delivery, although further studies are needed to advance our understanding of the role of PRRs during HDV infection.

### 2.2. Interferon Responses

Viral recognition by pattern recognition receptors activates the innate immune response. The net result of this recognition is the production of type I and type III interferons (IFN), including interferon IFN-α/β and IFN-λ, respectively [31]. After secretion from the cells, these IFNs bind to their cognate receptors present on the infected and noninfected cells, activating Janus kinases (JAKs), tyrosine kinase (TYK), signal transducer and activator of transcription (STATs) and interferon regulatory factor 9 (IRF9). The activation of these signaling pathways subsequently induces hundreds of IFN-stimulated genes (ISGs), employing antiviral activities [32]. The ability of HDV to trigger innate immune responses has been reported in HDV-infected cell lines, primary human hepatocytes and animal models [33,34,35]. Infection with HDV generates intense type I IFN and ISG responses in differentiated HepaRG cells, HDV-infected primary human hepatocytes, and sodium taurocholate cotransporting polypeptide (NTCP)-overexpressing HepG2 and HepaRG cells, where the virus replication is largely induced by IFN-β and IFN-λ [25,36]. IFN responses effectively restrain the early stages of HDV infection and control HDV RNA amplification during the hepatocyte proliferation phase [37]. In vitro transcriptome analysis of HDV-infected HepG2-NTCP cells confirmed the induction of several ISGs. NTCP-transgenic mouse studies and adeno-associated virus (AAV) transduction mouse models further confirmed the stimulation of HDV-induced type I IFNs and ISGs [24,38].

Cell division-mediated HDV spread has been observed in primary human hepatocytes-transplanted mouse models and the hepatoma cell line HepG2NTCP. This was further confirmed in Huh7NTCP cells, deficient in IFN response, showing profound HDV propagation through cell division, whereas such propagation was not seen in IFN-competent HepaRGNTCP cells [39]. Both HDV-induced IFN response as well as exogenous IFN treatment suppress cell division-mediated HDV spread, apparently through the acceleration of HDV RNA decay. This inhibitory capacity is more effective during cell division than the inhibition of intracellular HDV RNA replication in resting cells. The strong suppression of HDV during cell division by IFN response may result from increased accessibility of nucleus-resident HDV replication intermediates to cellular nucleases, including the ones induced by IFN during mitosis. HDV RNA stability assays in HepaRGNTCP and HepaRGNTCP-shMAD5 cells reinforced that the virus-induced IFN response destabilizes HDV RNA and possibly inhibits HDV RNA synthesis during cell division, while this effect is much smaller in the resting cells [39]. However, the identification of accountable ISGs and their mechanisms of action needs further investigation.

Current combination therapies, including Bulevirtide plus peg-IFN-α or Lonafarnib plus peg-IFNλ1, exhibited compelling synergistic anti-HDV effects in terms of immediate and strong decline in serum HDV RNA [40]. Of note, both Bulevirtide and Lonafarnib are not involved in controlling the cell division-mediated spread of HDV in vivo. Hence, the synergy monitored in this clinical combination treatment might be attributed to their joint inhibition of extracellular spread, while cell division-mediated HDV spread is targeted by peg-IFN-α and peg-IFNλ1. This suggests a novel mode of IFN action in controlling HDV replication and spread [39,41]. These results provided the basis for the development of combination therapies in terms of better outcomes. It is interesting to know that HDV-induced activation of the hepatic IFN system led to potent suppression of HBV, while HDV inhibition is moderate, which indicates that these viruses are equipped with different immunogenicity and sensitivity to the antiviral effectors of IFN, resulting in differential outcomes [42]. The persistent HDV-induced activation of hepatic IFN results in a tissue-wide state of IFN tolerance, ensuing therapeutic IFNs’ ineffectiveness. However, this could be overcome by developing a strategy that provisionally interrupts the state of IFN tolerance before starting the antiviral therapies that would help in improving treatment outcomes.

A recent study investigated the responsiveness of three HDV strains to peg-IFN-α in human liver chimeric mice infected with HBV and different strains of HDV [43]. The study demonstrated that the newly cloned HDV-1p and HDV3 strain strongly respond to peg-IFN-α treatment, observed by a decline in HDV viremia and intrahepatic HDV markers in mice, while a commonly used HDV-1 strain was unresponsive to IFN treatment, showing IFN resistance. The findings highlight that IFN responsiveness differs between different strains of HDV, suggesting heterogeneity among HDV strains. Though all these studies convince that HDV propagation prompts IFN responses, these responses are not as strong as seen in other viral infections. Also, these studies were mainly conducted in acute HDV infection settings where HDV viremia is usually high; therefore, further investigations in chronically infected patients are required, as HDV viremia really varies in these patients, which might affect the intensity of HDV-induced innate immune response.

### 2.3. JAK-STAT Signaling

Several pieces of evidence suggest the importance of Janus kinase/signal transducer and activator of transcription (JAK/STAT) signaling in human diseases by regulating immunological mechanisms to fight the infection and prevent disease progression. This signaling pathway is involved in several phenomena, such as orchestrating the immune system for cytokine secretion, the modulation of the T helper cell compartment, the activation of gene transcription and the regulation of downstream signaling in numerous molecules [44,45,46]. The activation of JAK-STAT signaling by IFNs leads to the upregulation of numerous ISGs, which then results in the killing of the virus-infected cells [47]. Due to its innate antiviral functions, this pathway is gaining much attention as a promising target to boost host innate immunity. However, an in-depth understanding of the JAK-STAT pathway is critical for designing new drugs that target diseases in which the JAK-STAT pathway plays a significant role.

The JAK-STAT signaling pathway functions through cellular receptors such as tyrosine kinase-related receptors and JAK and STAT proteins [48]. The JAK family consists of four members comprising JAK1, JAK2, JAK3 and TYK2 [49]. Binding cytokines to their specific receptors changes the conformation of JAKs present in the cytoplasm. This induces autophosphorylation or transphosphorylation in them, which subsequently phosphorylate STATs, initiating gene transcription in the nucleus, including several ISGs, providing protection against viral infections [50,51]. Alterations in the antiviral innate immune response have been projected as crucial cellular targets for the development of novel pan-viral therapeutic strategies. The JAK-STAT pathway is particularly significant due to its indispensable contribution to the regulation of localized and systemic inflammation against viral infections; therefore, it serves as a potential therapeutic target.

Recently, the role of JAK1 has been reported in the HDV life cycle, revealing an unexpected proviral function of JAK1 during HDV infection across multiple in vitro models, including primary human hepatocytes [52]. This proviral activity operates independently of the MDA5-mediated innate immune response and STAT3 activation. Inhibition or depletion of JAK1 with its inhibitor Upadacitinib suppresses HDV replication via the modulation of ERK1/2 signaling, and the phosphorylation levels of the small hepatitis delta antigen phosphorylation levels. The effect of Upadacitinib on HDV infection was assessed by treating the cells with the inhibitors for 24 h prior to viral inoculation and continuing treatment for 8 days post-infection. Intriguingly, only Upadacitinib, and not Fedratinib, prevented HDV infection in a dose-dependent manner. Upadacitinib adequately inhibited viral replication even when administered 3 days after infection at a concentration of 1 µM, indicating its ability to suppress HDV replication at various stages of post-inoculation. These findings suggest that JAK1-specific inhibitors like Upadacitinib could serve as promising antivirals for HDV treatment [52]. However, this would require rigorous and extensive investigations in in vivo models. Furthermore, given the immunosuppressive properties of JAK1 inhibitors, their interactions with IFN-cotreatment need to be investigated, as these drugs may accompany an increased risk of viral infections in patients. Overall, further investigations on JAK1′s role in HDV replication, disease progression and immunological functions could provide valuable insights for developing new therapeutic targets.

### 2.4. CD4+ T Cells

Induction of innate immunity primes adaptive immune cells, leading to the development of an effective antiviral immunity. Numerous human viruses, including hepatitis A-D, exhibit hepatotropism, preferentially infecting hepatocytes and causing immune-mediated liver inflammation. A robust and broad T cell response targeting multiple viral epitopes is required to clear the infection [53,54,55]. Growing evidence indicates that virus-specific CD4+ and CD8+ T cell responses play a critical role in competently eliminating the infection, killing the infected cells, inducing the production of neutralizing antibodies, inhibiting viral spread and clearing the extracellular virus particles [56,57,58,59]. However, the failure of these responses leads to viral persistence, which subsequently contributes to disease pathogenesis and severe outcomes in patients with chronic viral infections, highlighting defective adaptive immune response as a major determinant of viral persistence [60]. Nevertheless, there is an inadequate understanding of virus-specific T cell responses during chronic hepatitis D (CHD) infection. The findings that HDV transgenic mice (expressing both small and large HDAg) exhibit no signs of cytopathology nor develop any form of liver disease indicate that HDV is not directly cytopathic [61]. In fact, HBV carriers experiencing rapid disease progression and severe course of liver disease after HDV superinfection, along with significant intrahepatic infiltration of mononuclear cells, suggest that liver injury in HDV infection is likely driven primarily by immune-mediated mechanisms. It has been reported that during HDV infection, T cells can recognize different antigenic determinants; however, this recognition varies between patients depending on their individual responses to specific peptides [62].

Very few studies have investigated HDV-specific CD4+ T cells in CHD infection. Since viral infections may alter the frequencies and phenotypes of immune cells, a study tested whether HDV infection affected the total T cell population. The results showed that HDV infection does not alter the frequencies of CD3+, CD4+ and CD8+ T cell subsets [63]. However, the capacity of T cell proliferation and cytokine production against HDVAg was hampered [64], as shown by reduced IFN-γ and TNF-α production by both CD4+ and CD8+ T cells. Notably, loss of proliferative response was not associated with any of the clinical parameters. Another study revealed that CD4+ T cells can target 18 different HDAg regions [64,65]; nonetheless, these cells are only visible after antigen-specific culture and possess poor viral-specific responses. Although patients with acute HDV infection exhibited detectable CD4+ T cells under ex vivo conditions, along with robust cytokine production similar to that observed in HBV and HCV infections, this indicates that strong CD4+ T cell responses do exist during acute HDV infection but gradually decline over time as the disease progresses to CHD infection [65]. A study by Nisini et al. detected HDV-specific CD4+ T cell responses in patients with normal alanine aminotransferase (ALT) levels [64], which was contradicted by another study showing no association between HDV-specific CD4+ T cell responses and ALT levels [65]. Remarkably, during CHD infection, the number of global CD4+ T cells increases with advanced liver disease, and these cells exhibit stronger cytotoxic potential than those of HBV and HCV infection [66]. Moreover, these cells display the phenotype of terminally differentiated effector cells, lacking the expression of CD28 and CD27. Furthermore, the cytotoxic ability in these cells is primarily attributed to either Th0 or Th1 subsets, indicating the prevalence of these subsets during CHD infection. Data obtained from human leukocyte antigen (HLA)-binding assays revealed the presence of several HLA class II-restricted epitopes on CD4+ T cells [64]. However, the contribution of CD4+ T cells to HDV infections needs further investigation.

### 2.5. CD8+ T Cells

Sequential activation of various immune cells, including CD8+ T cells, occurs during viral infection. Resting naïve CD8+ T cells have an astonishing capacity to respond to pathogens by colossal expansion and differentiation into cytotoxic effector cells and clear the infection. The role of CD8+ T cells in HBV infection is well documented [58,67]. These cells respond to infection through their T cell receptors to distinct HBV antigens presented by MHC class I molecules and get activated. After activation, CD8+ T cells secrete cytokines, chemokines and cytotoxic granules that directly lyse the infected cells [68]. These cells are the main effectors liable for viral clearance during acute infection; however, in the case of chronic infection, they lose much of their effector function and can partially contribute to viral control. Rather, these cells participate in immune-mediated pathogenesis and promote disease progression in chronic HBV infection [60].

The role of CD8+ T cells is not well studied during HDV infection. However, a study reported a reduction in HDV-specific CD8+ T cells in HDV-infected patients [63]. The majority of these HDV-specific CD8+ T cells were PD-1+ and CD127+, constituting a memory phenotype, mostly effector memory cells. The remaining proportion typically exhibited central memory cells and a small population of terminal effector memory cells. The frequencies of CD8+ T cells are associated with transaminase activity, inferring that these cells contribute to HDV pathogenesis. Conversely, HDV-specific IFN-γ secretion by these cells inversely correlates with HDV titer, indicating the importance of HDV-specific CD8+ T cell response in viral decline [63]. CD8+ T cells can recognize HDV epitopes presented by multiple HLA molecules, with a subset of activated HDV-specific CD8+ T cells targeting conserved viral epitopes. However, half of the HDV-specific CD8+ T cells have a memory-like phenotype displaying PD1+CD127+TCFhighT-betlow, where high TCF expression is associated with lack of in vivo stimulation with their cognate antigen. This indicates that HDV developed escape mutations to elude HDV-specific CD8+ T cell recognition in chronic infection. The study further demonstrated that despite being small, HDV is very immunogenic, and disease progression is possibly due to immunopathogenesis. A small number of resolved HDV patients showed the presence of HDV-specific CD8+ T cell responses, while no change in proliferative capacity was seen between resolved HDV infection and chronic HBV/HDV coinfection [69,70]. Moreover, the intrahepatic profile of CD8+ T cells revealed the presence of memory and terminal effector memory cells and barely constituted any naïve population [71]. The effector memory cells displayed markers of tissue-resident cells (CD69+CXCR6+) and expressed CD38 and CD107a. However, these cells were exhausted and exhibited PD1 and CD39 expressions and had NKG2D expression, which is associated with TCR-independent bystander activation. On the contrary, peripheral CD8+ T cells had most of the naïve population, followed by terminally differentiated effector cells and memory cells. Additionally, blood memory T cells constituted a unique population that was CD127+PD1+CD38+CD39+NKG2D+ and were not present in the liver, suggesting the presence of more heterogeneity in circulating cells than those in the intrahepatic compartment. Interestingly, the phenotype of circulating HDV-specific and total CD8+ T cells was almost similar in terms of their activation and exhaustion. While circulating and intrahepatic HDV-specific and total CD8+ T cells represented different phenotypic characteristics in terms of both activation and exhaustion. Intrahepatic CD8+ T cells were overly activated, exhausted, and had higher degranulation capacity than those of cells in circulation. Overall, the study suggests that the activation of antigen-nonspecific liver-resident CD8+ T cells may subsidize inflammation and disease progression during HDV infection [71].

### 2.6. Role of Inhibitory Receptors

Various conserved negative regulators of T cell activation function as checkpoint inhibitors to calibrate the immune response and prevent hyperactivation [72]. Among several inhibitory molecules, programmed death-1 (PD-1) and cytotoxic T lymphocyte antigen 4 (CTLA-4) are the most compelling T cell immune checkpoint molecules. These molecules employ their biological functions during the T cell lifespan, complementing each other’s functionality by ensuring that T cells maintain self-tolerance while efficiently shielding the body from viral pathogens. During chronic viral infection, persistent T cell exposure to antigen or inflammatory signals drives T cell exhaustion, a condition characterized by the sustained expression of numerous inhibitory receptors, such as PD-1 and CTLA-4 on T cells, accompanied by the loss of effector functions and memory T cell properties, restricting viral control [73,74]. T cell exhaustion was initially identified in mice with chronic viral infection and was later perceived in humans against hepatitis C and HIV infection [75,76].

The manifestation that T cell exhaustion is reversible provides considerable clinical opportunities to block the inhibitory molecules for the recovery of T cell functions. Therefore, in the past few years, PD-1 and CTLA-4 have been successfully targeted in various ground-breaking research for a wide variety of disease conditions, such as chronic viral infections and cancers, to reverse this dysfunctionality and reinvigorate immune responses [77,78,79]. Recently, it has been shown that CD8+ T cells in HDV-infected patients show an exhaustive phenotype by expressing 2B4, CD160 and PD-1. HDV-specific effector memory CD8 T cells expressed higher PD-1 expression in comparison to other viral infections, such as CMV and EBV, while the coexpression of all three exhaustion markers was lower among these cells [63]. Importantly, the in vitro blockade of PD-1 and CTLA-4 pathways improved HDV-specific CD4+ T cell responses in 46% and 23% of the patients, respectively [80]. While PD1 blockade intensified the strength and breadth of the CD4+ T cell response, CTLA-4 inhibition could not contribute much. The simultaneous blockade of PD-1 and CTLA4 did not show any additional benefit. In fact, the responses achieved after the PD-1 blockade declined by additional blocking with CTLA-4. Distinctly from CD4, CD8+ T cells minimally achieved functional restoration post PD-1 and CTLA-4 blockade, proposing that other factors might be contributing to functional exhaustion in CD8+ T cells. Also, this might indicate that CD4+ T cells are more sensitive to manipulation against blockade of inhibitory pathways, rather than CD8+ T cells. Intriguingly, the addition of IL-12 further increased the functionality of T cells in terms of IFN-γ and TNF-α secretion. Moreover, IL-12 stimulation broadened and strengthened the capacity of cytokine-producing T cells, indicating that a combination of checkpoint inhibitors and IL-12 cytokine may effectively reverse T cell exhaustion during HDV infection [80]. Overall, the effect of HDV on the immune system has been summarized in Table 1.

## 3. HDV in Liver Disease Progression and Oncogenesis

Hepatocellular carcinoma (HCC) remains the sixth most common cancer globally and the second leading cause of cancer-related death [83,84]. Persistent HDV replication and hepatic inflammation turn out to be the culprits of cirrhosis and HCC development, where active replication with both HBV and HDV further accelerates disease progression, leading to early cirrhosis and HCC [85]. While chronic infection with both viruses results in a higher incidence of HCC development, not all persistently infected individuals develop liver cancer, giving us a clue that the cancer development is a multifactorial process. The virus alone is not sufficient to lead to cancer development and offers only a slice of oncogenic alterations, indicating that the amalgamation of both viral and host factors is needed for cancer development [86]. Viral infections have their exclusive molecular mechanisms of carcinogenesis by inducing gene mutations in hepatocytes and triggering their transformation along with the induction of chronic liver inflammation that closely relates to the HCC manifestations [87].

A recent comprehensive meta-analysis supported the fact that CHD infection is associated with an increasing risk of HCC than HBV monoinfection; however, the mechanism by which HDV induces HCC has not been entirely defined [88]. HDV might contribute as a crucial etiological factor promoting serious liver diseases such as fulminant hepatitis, cirrhosis, HCC, and, in fact, premature deaths. However, the course of disease is variable and heterogenous in different parts of the world. Multiple studies from European countries claimed the association of HDV with increasing risk of HCC, where one study showed nearly three-fold higher risk of HCC in patients coinfected with HBV and HDV, while the other reported six-fold higher risk of HCC in comparison with HBV infection alone, suggesting HDV is directly oncogenic [89,90,91]. A 28-year follow-up study established that persistent HDV infection encourages cirrhosis and HCC at rates of 4% and 2.8% annually [92]. Still, the interpretation of these data needs a cautious approach. In the meta-analysis by Alfaiate et al., the difference in HCC development between HBV and HDV became evident in studies after 2000 and in particular after 2010, when HBV patients were treated with nucleos(t)ide analogs [88]. In a study assessing the outcome of patients who responded to management of CHD with an INF treatment regimen, there was a trend not to develop HCC among patients with viral response to treatment [2]. These data suggest that not HDV per se but the lack of an efficient treatment against HDV may be an important risk factor for HCC development. On the other hand, HDV can alter crucial signaling pathways related to fibrosis, such as epigenetic modifications, dysregulations of long-noncoding RNAs (IncRNAs), proteomic changes, and significant immunological alterations that trigger fibrosis [93,94,95,96]. In addition, CHD infection promotes the infiltration of inflammatory cells, which helps in creating and maintaining the microenvironment required for HCC development [97].

Analogous to HBV, HDV-mediated liver damage is also predominantly an immune-mediated process. HDV can induce a strong innate immune response after being recognized by pattern recognition receptors such as MDA5, implying that HDV infection can change the inflammatory profile in HBV-infected patients. Persistence and a higher degree of inflammation in the liver may reflect accelerated fibrosis in these patients; however, the mechanism by which HDV alters the immune system is different than that of HBV and HCV infection due to the constant presence of coinfection. HDV large antigen L-HDAg has oncogenic properties by upregulating transforming growth factor-β (TGF-β) mediated transcriptional activity of C-Jun that modulates cell growth, proliferation, and apoptosis [98]. A similar signaling cascade is triggered by the interaction between HBx and Smad protein that was further enhanced by L-HDAg in a dose-dependent manner. These synergistic molecular mechanisms partly shed light on why HDV/HBV dual infection is associated with a higher incidence of HCC than HBV monoinfection. Augmentation of TGF-β signaling has been observed in HBV/HDV-coinfected patients that can accelerate cellular growth and differentiation and can play a central role in inflammation, fibrogenesis and immunomodulation in the HCC microenvironment [99]. L-HDAg can also trigger the TGF-β pathway through the Smad3 protein and other oncogenic pathways, comprising nuclear factor kappa (NF-κB) through tumor necrosis factor-α (TNF-α) activation and oxidative stress. Further, L-HDAg may trigger the JAK-STAT pathway through the activation of STAT3 or c-FOS activation [98]. Additionally, other mechanisms of HDV-related oncogenesis include the downregulation of a tumor suppressor gene, glutathione S-transferase P1 (GSTP1), which was evidenced by the transfection of a fetal hepatic cell line with S-HDAg that restrained GSTP1 expression specifically by binding to its mRNA, resulting in the accumulation of ROS and enhanced apoptosis. Recently, microarray-based data comparing cancerous and para-cancerous specimens from subjects with CHB- and CHD-related HCC found seven differentially expressed genes (CDC6, CDC45, CDCA5, CDCA8, CENPH, MCM4 and MCM7) only in the CHD-driven HCC subgroup, and these genes were closely involved in mitotic cell cycle and DNA replication, suggesting these pathways were selectively mediated by HDV [100]. Altogether, these findings show that the molecular profile of CHD-related HCC is defined by an overexpression of genes involved in cell cycle and DNA replication/repair, emphasizing genomic instability as a key mechanism of hepatocarcinogenesis.

Since HBV is consistently present in all HDV-infected patients, the interaction between HDV and HBV could also encourage HCC development. Long-term studies have reported extended and complex interplay between HDV and HBV with variable levels of viremia [101]. More than 50% of cases display HBV activity at different time points, relating it to poor prognosis. HBV and HDV jointly infect the cells via numerous mechanisms and induce abnormal regulation of cell signaling pathways by their viral proteins, including HBx, L-HDAg and S-HDAg, directing the occurrence of HCC [102]. HBV proteins, mainly HBx, play a crucial role in HBV life cycle and participate in HCC development by several mechanisms such as integration into the hepatocyte genome endorsing genetic instability, activation of cell survival signaling pathway and inactivation of tumor suppressors, epigenetic modifications such as DNA methylation, histone acetylation, microRNA expression and interaction with mitochondrial and other cellular proteins to induce oxidative stress [103,104,105,106].

## 4. Considerations for Future Immunological Studies

While various new compounds are currently in development to treat and slow the progression of the HDV infection, unfailing surrogate markers to predict sustained suppression of HDV following cessation of antiviral therapy are not defined. Critical gaps persist in our understanding of the immune correlates associated with viral clearance and future protection. Future immunological studies are required to address present unanswered questions that might include

1. What are the specific immune determinants/correlates that drive HDV clearance in chronically infected patients?
2. Do these immune correlates differ between patients who clear the virus spontaneously and those who achieve clearance through antiviral therapy?
3. Does HDV clearance induce a distinct immune profile that reduces the risk of fibrosis progression or hepatocarcinogenesis?
4. Can immune correlates be used to guide the safe discontinuation of antiviral therapy in chronically infected patients?

Addressing these questions may help define the immune signatures associated with HDV clearance and inform the development of innovative therapeutic approaches, potentially combining antiviral agents with host-targeted immunotherapeutic strategies.

## 5. Conclusions

Induction of innate immune pathways triggering IFN signaling might be crucial in the development of antiviral immunity, resulting in the resolution of HDV. However, HDV induces several changes in the immune system, such as promoting JAK signaling to support viral replication, the upregulation of the inhibitory receptors on T cells to induce immune exhaustion and impairment in effector T cell function, and enabling viral persistence. A comprehensive analysis of host immune responses in HDV-infected patients will enhance our understanding of HDV immunopathogenesis and further help uncover potential immunotherapeutic pathways essential to achieve a functional cure for HDV infection.

## Figures and Tables

**Table 1:** HDV-induced changes in the immune system.

Effect	Model	Outcome	Reference
Induction of type I IFNs and ISGs	In vitro culture system (HepaRG cells, HDV-infected primary human hepatocytes (PHH), NTCP-overexpressing HepG2 HepaRG, and Huh7^NTCP^ cells) In vivo mouse models	Controls early stages of HDV infection, cell division and HDV RNA amplification	[24,25,28,35,37,38,39,42]
Activation of JAK/STAT pathway	Huh7-NTCP, HepG2-NTCP PHH	Promote HDV replication	[52]
Decreased frequencies of MAIT and NK cells, retained activation and degranulation capacity	Human PBMCs, intrahepatic immune cells	Progressive loss of function	[71,81]
Increase in global CD4+ T cells with strong cytotoxic potential	Human PBMCs	T cell dysfunction/inflammation	[64]
Reduction in CD4 T cell proliferation	Human PBMCs	T cell dysfunction/viral persistence	[64]
Weaken Th1 and Th2 cytokine responses	Human PBMCs		[82]
Reduction in HDV-specific CD8+ T cells	Human PBMCs and intrahepatic immune cells	Viral decline and immunopathogenesis	[63]
Induction of global CD8+ T cells	Human intrahepatic immune cells	Inflammation associated with disease progression	[71]
CD8+ T cell exhaustion and activation	Human intrahepatic immune cells	T cell dysfunction/viral persistence	[71]

## Data Availability

Not applicable.
